# A case of vestibular schwannoma mimicking burning mouth syndrome

**DOI:** 10.1186/s13030-021-00209-y

**Published:** 2021-03-21

**Authors:** Takayuki Suga, Miho Takenoshita, Trang T. H. Tu, Takashi Sugawara, Susumu Kirimura, Akira Toyofuku

**Affiliations:** 1 Department of Psychosomatic Dentistry, Graduate School of Medical and Dental Sciences, Tokyo Medical and Dental University, 1-5-45 Yushima, Bunkyo-ku, 113-8510 Tokyo, Japan; 2 Department of Neurosurgery, Graduate School of Medical and Dental Sciences, Tokyo Medical and Dental University, Tokyo, Japan; 3 Division of Surgical Pathology, Tokyo Medical and Dental University Hospital, Tokyo, Japan

**Keywords:** Burning mouth syndrome, Vestibular schwannoma, Brain tumor

## Abstract

**Background:**

An oral burning sensation with unidentified cause in patients with preexisting psychosocial conditions is usually diagnosed as burning mouth syndrome. However, unexpected organic lesions may be detected in rare cases.

**Case presentation:**

A 35-year-old woman had chief complaints of a burning sensation and numbness of the right side of the lip and tongue, as well as a dry sensation of the mouth with a taste disturbance of the right side of the tongue. The symptoms were continuous and did not show any daily fluctuations. The symptoms started without any recognizable triggering factor six months before her first visit to our clinic,. No abnormality was detected in her mouth. MRI images revealed an approximately 30 × 30 mm well-defined mass localized in the right cerebropontine angle compressing the trigeminal nerve, which was diagnosed as schwannoma of the right auditory nerve.

**Conclusions:**

It is important for clinicians to consider the possibility of brain tumors in their differential diagnosis of BMS. Although it is not always easy to eliminate all diseases that may cause an oral burning sensation in patients with BMS-like symptoms, more attention and careful examination based on the patient’s psychosomatic background features and other possible causes are needed to rule out organic diseases.

## Background

Burning mouth syndrome (BMS) is a recurrent burning pain without evident cause. Patients with BMS often have accompanying taste disorders and dry mouth. The prevalence of BMS in the normal population ranges from 1.5 to 10 %. Middle-aged women are most commonly affected, especially after menopause [1]. An oral burning sensation with unidentified cause in patients with psychosocial factors tends to be diagnosed as BMS. However, unexpected organic lesions may be detected in rare cases. Here, we present a case of Vestibular schwannoma (VS, acoustic neuroma) with BMS-like symptoms and discuss the difficulties in finding the cause of the oral burning.

## Case presentation

**Fig. 1:**
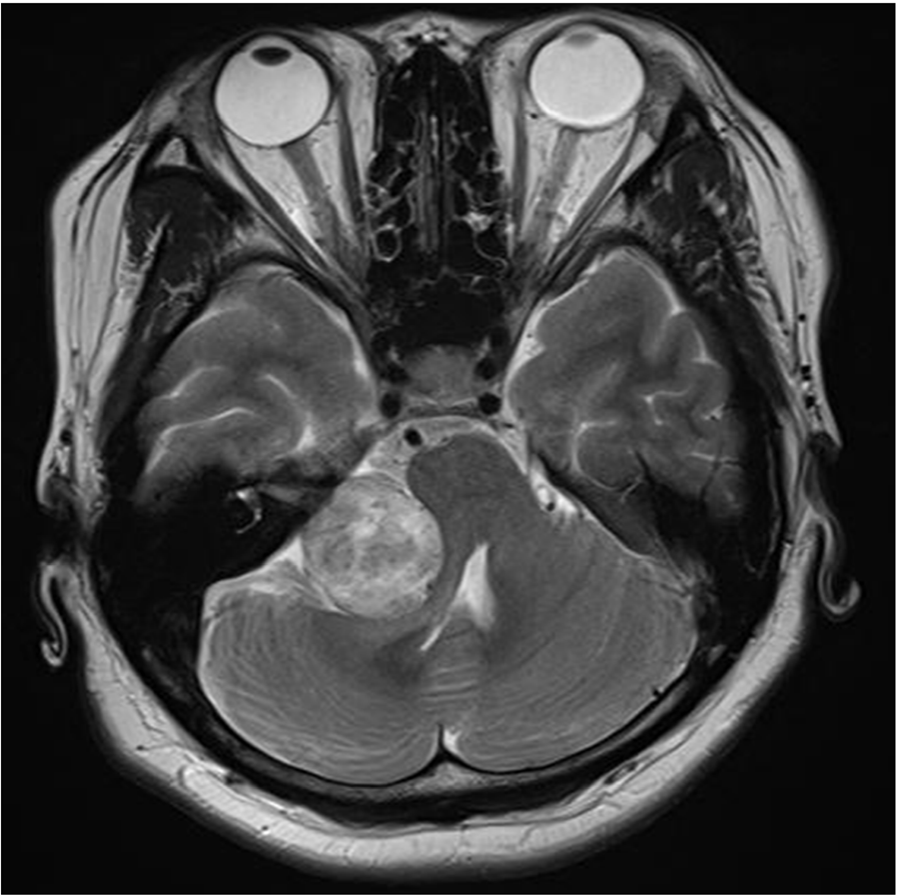
MRI image. Axial magnetic resonance image shows a 30 mm×30 mm well-defined mass involving the right cerebellopontine angle

**Fig. 2:**
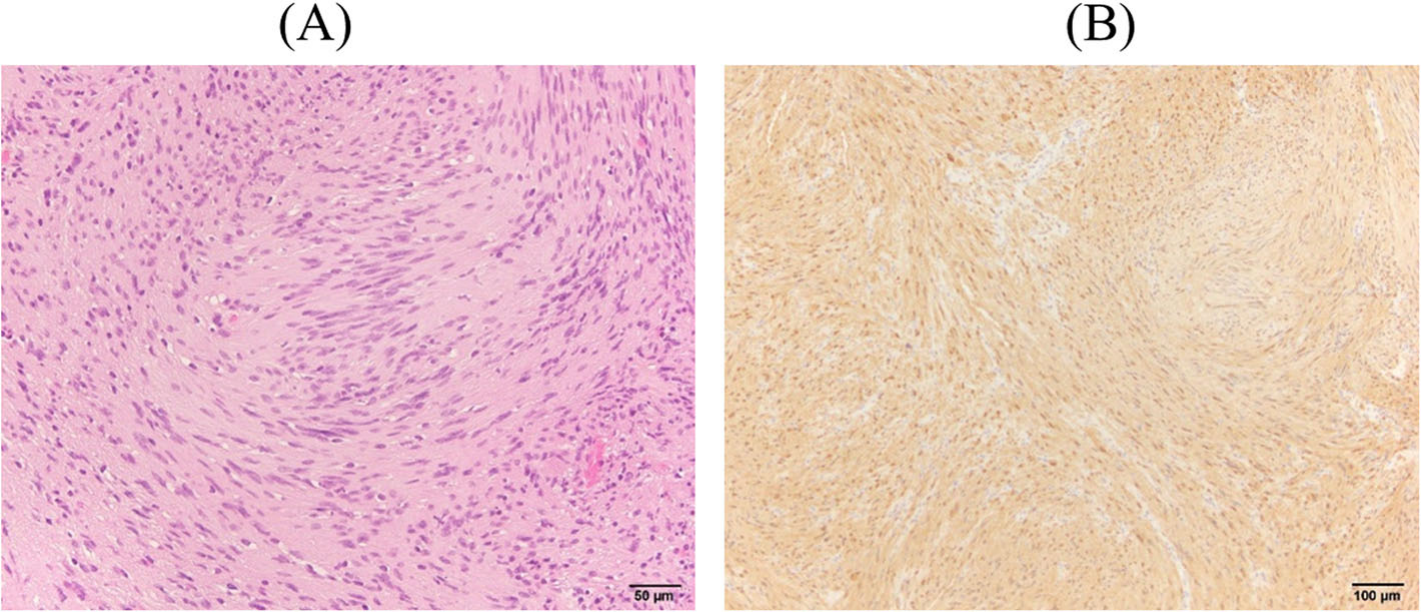
Pathological image. **a** Hematoxylin & eosin (H&E) stain with ×200 power showing a palisading pattern of the schwannoma, Antoni A. **b** S-100 protein immunohistochemical stain with ×100 power

A 35-year-old woman had chief complaints of a burning sensation and numbness of the right side of the lip and tongue, as well as a dry sensation of the mouth with a taste disturbance of the right side of the tongue. It did not present sharp, shooting, and shock-like pain. These symptoms were predominant on the right side. The symptoms were continuous and did not show any daily fluctuations. Even though she presented numbness on the tongue, she could move it smoothly. Her speech was not affected by the burning sensation. The patient’s medical history included uterine fibroids, irritable bowel syndrome, and migraine. In her twenties, she visited a psychiatrist but the diagnosis was unclear. Six months before her first visit to our clinic, the symptoms started without any recognizable triggering factor. She also visited the oral surgery department in a general hospital, where she was prescribed xylocaine jelly and carbamazepine 200 mg /day. However, her symptoms were not relieved. Because the patient had irritable bowel syndrome, insomnia, and a busy and stressful life, she was referred to the psychosomatic dental clinic in Tokyo Medical and Dental University Dental Hospital. At her first visit, we conducted a structured medical interview and intraoral examination, but no abnormality was detected. Allodynia, ulcer, and swelling of the tongue were absent. Herpes infection was excluded by antibody testing. The visual analog scale score for pain intensity was 69, and the Zung’s self-rating depression scale score was 48, which was almost the upper normal limit. For the neurological testing, Semmes-Weinstein monofilament testing on the region of second and third branch of the trigeminal nerve also presented no sensory abnormality. Unlike the typical feature of BMS, food intake did not ease the symptoms. Therefore, magnetic resonance imaging (MRI) examination was scheduled to rule out intracranial causes. MRI images revealed an approximately 30 × 30 mm well-defined mass localized in the right cerebropontine angle compressing the trigeminal nerve, which was diagnosed as schwannoma of the right auditory nerve (Fig. 1). The additional symptoms besides pain described may have been due to the tumor compression affecting cranial nerve VII. At this point, we referred the patient to a neurosurgeon who confirmed that the patient had been suffering from hearing loss for three months. The tumor was successfully removed by surgery. Hematoxylin and eosin staining showed spindle cells and a fasciculated, palisading pattern (Fig. 2). The cells showed positivity to S-100 protein. The diagnosis of schwannoma of the right auditory nerve was confirmed. Though her facial paralysis showed slight improvement, the patient’s oral burning sensation and numbness persisted for 1.5 years after the surgery. While the patient could not perceive sour and salty tastes, she reported vague perception of sweet and bitter ones.

## Discussion

A burning sensation in the mouth can be caused by many factors [1]. Although the etiology of BMS suggests that psychosocial factors play an important role, in the case of patients with preexisting psychosocial conditions clinical misdiagnosis is possible if the possibility of brain tumor is overlooked.

VS is a rare benign tumor that develops in the acoustic nerve Schwann’s sheath [2]. The incidence of VS is estimated to be 1.2 per 100,000 of the population, and the median age was 55 years. Common symptoms of VS are hearing loss, tinnitus, and vertigo, which tend to be regarded as unidentified complaints or medically unexplained symptoms.

About 10 % of patients with VS have additional complaints of atypical symptoms like orofacial pain or paresthesia. In some cases of VS, the patients present with orofacial or tooth pain and are referred to a dentist. Trigeminal neuralgia secondary to VS is often reported. Owing to proximity to the trigeminal nerve, VS sometimes causes trigeminal nerve symptoms. However, VS accompanied by symptoms mimicking BMS is rarely reported [3].

There are several reports of chronic oral pain arising from tumors in the craniofacial region. We have limited experience with oral burning caused by brain tumors. In our case, although the patient had unilateral symptoms, the characteristics of the symptoms, such as taste disturbance and non-shock like pain, were different from those of trigeminal neuralgia. Our patient’s symptoms, medical history, comorbidity of irritable bowel syndrome, migraine, busy and stressful lifestyle, sleep disturbance, and history of visiting a psychiatrist implicated the characteristics of BMS. Pikoff states that psychological mislabeling occurs when anxiety, depression, and stress are assumed to be related to poorly understood chronic pain [4]. The atypical characteristics for BMS in our patient was laterality of the symptoms, a relatively young age for BMS, non-remission by food intake, and absence of daily fluctuations of burning sensation. Table 1 shows a comparison of the characteristics of VS and BMS. The prevalence of taste disturbance is reported to be 11–69 % in patients with BMS and approximately 30 % in patients with VS. In addition to taste disturbance, VS and BMS have other common characteristics, such as trigeminal nerve dysfunction including chronic oral pain. Careful neurological tests or MRI can provide sufficient information to rule out brain tumors such as VS. Therefore, ancillary examination maybe helpful in young patients with unilateral and atypical symptoms for BMS.

It is important for clinicians to consider brain tumors in their differential diagnosis of BMS. In patients with BMS-like symptoms easily regarded as psychogenic, it is not easy to eliminate all diseases that may cause an oral burning sensation, thus more attention and careful examination are needed to rule out organic disease.

**Table 1:** The comparison of the characteristics in burning mouth syndrome and vestibular schwannoma

	Burning mouth syndrome	Vestibular Schwannoma
Laterality	mostly bilateral	unilateral
Burning sensation	Yes	Yes
Daily fluctuation	Yes	No
Taste disorder	69%(Grushka 1987)	30 - 40.8% (Watanabe 2003, Sahu 2008)
Trigeminal nerve dysfunction	Yes (Jaaskelainen 2017)	Yes (Selesnick 1993)
Highest prevalence	60 - 69years (Bergdahl 1999)	52 - 55years (Propp 2006)
Neurological abnormality	No	Yes
Hearing loss	No	Yes

## Data Availability

The dataset supporting the conclusions of this article is available in from the Department of Psychosomatic Dentistry, Graduate School of Tokyo Medical and Dental University.

## Abbreviations

BMS: Burning mouth syndrome
VS: Vestibular schwannoma
MRI: Magnetic resonance imaging
